# The marine-derived HIF-1α inhibitor, Yardenone 2, reduces prostate cancer cell proliferation by targeting HIF-1 target genes

**DOI:** 10.1186/s11658-024-00617-2

**Published:** 2024-07-08

**Authors:** Siyong Peng, Yingbo Guo, Marie Irondelle, Abigail Mazzu, Michel Kahi, Paula Ferreira Montenegro, Frédéric Bost, Nathalie M. Mazure

**Affiliations:** 1 Université Côte d’Azur, Institut National de la Santé et de la Recherche Médicale, Nice, France; 2 Inserm U1065, Centre Méditerranéen de Médecine Moléculaire (C3M), Equipe Labellisée Ligue Nationale Contre le Cancer, Nice, France; 3 Institut de Chimie de Nice, Université Côte d’Azur, CNRS UMR 7272, 06108 Nice, France

**Keywords:** Docetaxel, HIF-1 inhibitor, Hypoxia, Marine microenvironment, Microtubules, Prostate cancer, Yardenone

## Abstract

**Background:**

Prostate cancer (PCa) ranks as the second most prevalent cancer in men, with advanced stages posing significant treatment challenges. Given its solid tumor nature, PCa is highly susceptible to hypoxia, a condition associated with resistance to radiation and chemotherapy, metastasis, and unfavorable patient outcomes. Hypoxia-inducible factors (HIFs) play a pivotal role in cancer cell adaptation to hypoxic environments, contributing to treatment resistance. Consequently, inhibitors targeting HIFs hold promise for cancer therapy.

**Methods:**

In this study, we aimed to characterize novel HIF-1α inhibitors including Sodwanones A (1), B (2), C (3), G (4) and Yardenone 2 (5) isolated from marine sponges belonging to the *Axinella genus*. Our investigation evaluated the impact of these compounds on various aspects of HIF-1α regulation, including stabilization, nuclear localization, expression of HIF-1 target genes (while sparing HIF-2 target genes), cellular metabolism, as well as cell proliferation and viability in prostate cells under hypoxic conditions.

**Results:**

Our findings revealed that among the compounds tested, Yardenone 2 exhibited notable effects in hypoxia: it destabilized HIF-1α at the protein level, decreased its nuclear localization, selectively altered the expression of HIF-1 target genes, and restrained cell proliferation in aggressive PC3 prostate cancer cells as well as in an MSK-PCa3 patient-derived organoid line. Moreover, it affected the morphology of these organoid. Yardenone 2 was also compared to Docetaxel, a specific microtubule inhibitor and a drug used in the treatment of prostate cancer. The comparison between the two compounds revealed notable differences, such as a lack of specificity to hypoxic cells of Docetaxel.

**Conclusion:**

These results mark the first demonstration that Yardenone 2 functions as a cytostatic-like inhibitor impacting microtubules, specifically targeting hypoxic cancer cells. This discovery suggests a promising avenue for novel therapeutic interventions in prostate cancer.

**Supplementary Information:**

The online version contains supplementary material available at 10.1186/s11658-024-00617-2.

## Introduction

Hypoxia-inducible factor-1 (HIF-1) is a critical transcription factor that plays a central role in cellular responses to low oxygen levels, known as hypoxia [1–3]. HIF-1 orchestrates adaptive mechanisms to enhance oxygen delivery and cellular survival under hypoxic conditions. HIF-1 is a heterodimeric protein composed of an oxygen-sensitive α-subunit and a constitutively expressed β-subunit. Under normoxic conditions, prolyl hydroxylase enzymes hydroxylate specific proline residues on the α-subunit, marking it for ubiquitin-mediated degradation. However, in hypoxic environments, this hydroxylation is inhibited, leading to the stabilization and translocation of HIF-1 to the nucleus. Once in the nucleus, HIF-1 binds to hypoxia-response elements in target genes, promoting the transcription of various factors involved in angiogenesis, glycolysis, and cell survival.

However, dysregulation of HIF-1 has been implicated in various pathological conditions, including cancer, inflammation, and ischemic diseases. Given the pivotal role of HIF-1 in promoting tumor growth, invasion, and resistance to therapies, there is significant interest in developing small molecules that can selectively inhibit HIF-1 activity. Consequently, the development of HIF-1 inhibitors has emerged in the late 1990s as a promising avenue for therapeutic interventions [4, 5]. Compiling an exhaustive list of HIF-1α inhibitors is challenging due to the sheer volume of research and the continuous development of new compounds. Promising inhibitors such as epoxyquinomicin A, isolated from Streptomyces; YC-1, originally a soluble guanylate cyclase (sGC) stimulator; PX-478; topotecan, initially a topoisomerase I inhibitor; digoxin, a traditional cardiac medication; KC7F2; apigenin, a natural flavonoid; chloroquine, an antimalarial drug and cefepime, a beta-lactam antibiotic, have been found to inhibit HIF-1 and to modulate HIF-1-mediated gene expression. Their potential therapeutic uses have been carefully evaluated but all these compounds also affect other cellular and metabolic pathways. Certain compounds, including PX-478 and digoxin, entered Phase 1 trials without notable success, unlike HIF-2 inhibitors like PT2385 (https://clinicaltrials.gov/study/NCT03216499) or PT2977, also known as belzutifan, which demonstrated more promising outcomes [6].

The fascination with exploring marine microenvironments for new medical compounds stems from the unparalleled biodiversity and distinctive biochemical characteristics found in the world's oceans [7–10]. These environments, with a myriad of life forms ranging from microscopic organisms to larger marine species, present a vast and relatively untapped source of potential therapeutic agents. Marine organisms have evolved complex chemical strategies to adapt and thrive in diverse and often extreme habitats. The unique challenges posed by these environments have led to the development of bioactive compounds with novel structures and functions. The extreme conditions prevalent in marine microenvironments, such as high pressure, low temperature, deep-sea environments, and low oxygen concentrations contribute to the production of compounds with unusual properties. From a medical perspective, marine-derived compounds show promise in addressing challenges such as drug resistance, with antimicrobial agents being of particular interest. Additionally, the anti-cancer and neuroprotective properties demonstrated by some marine compounds offer potential avenues for novel drug development in these therapeutic areas [11, 12]. In this context, the exploration of HIF-1α inhibitors derived from the sea holds significant promise. In 1993, chemists identified three triterpenoids, Sodwanone A-C, from the sponge *Axinella weltneri* [13]. Subsequently, additional small molecular compounds like Yardenone were extracted from the sponge, and some demonstrated cytotoxic properties [14, 15]. Dai and colleagues reported eleven Sodwanone/Yardenone triterpenoids, examining their ability to inhibit HIF-1 activation through a luciferase reporter assay, indicating the potential role of these unique triterpenoids in HIF-1 inhibition [16].

This study aimed to investigate the impact of select Sodwanones/Yardenones sourced from the natural Sodwanones A (**1**), B (**2**), C (**3**), E/M, G (**4**), and Yardenone 2 (**5**) substances collection of Pr. M. Mehiri at the Nice Institute of Chemistry. Pr M. Mehiri’s group re-isolated the previously reported compounds Sodwanones A-C (**1–3**) and G (**4**) from *Axinella weltneri* collected in Sodwana Bay, South Africa and Yardenone 2 (**5**) from *Axinella cf. bidderi* from Yemen’s Socotra Island in the Indian Ocean as described in [17]. We conducted an initial screening on prostate cancer cell lines involving Sodwanone A, B, C, E/M, and G, as well as Yardenone. A subsequent assessment was carried out on another natural substance, Yardenone 2. Mechanistically, our study demonstrate that Yardenone 2 reduces proliferation in PC3 cells by directly effecting HIF-1 target genes, which is turn has a subtle impact on microtubule organization. A comparison with Docetaxel, a specific inhibitor of microtubule depolymerization, revealed that Yardenone 2, despite exerting a similar effect on microtubules, maintained its efficacy under hypoxic conditions, whereas Docetaxel proved to be less effective.

Despite the challenge posed by the limited quantity of each substance, the endeavor to identify a natural compound capable of influencing HIF-1α activity appears to have met with success.

## Methods

### Cell lines

The P69 cell line was derived by immortalization of human primary prostate epithelial cells with simian virus-40T antigen and was grown in RPMI 1640 with 10% FBS [18]. The prostate cancer DU145 (HTB-81) and PC3 (CRL-1435) cell lines, were purchased from the American Tissue Culture Collection and grown in the Dulbecco's modified eagle medium (DMEM) with 10% fetal bovine serum (FBS) and 1% penicillin/streptomycin antibiotics. The clear cell Renal Cell Carcinoma (ccRCC) 786-O (CRL-1932) cell line is part of the personal collection of Dr. N. Mazure and came originally from the laboratory of Dr. J. Pouysségur. Cells were incubated at 37 °C and 5% CO_2_. An INVIVO_2_ 200 anaerobic workstation (Ruskinn Technology Biotrace International Plc) set at 1% O_2_, 94% N_2_ and 5% CO_2_ was used for hypoxic conditions.

### Patients-derived organoid lines, MSK-PCa3

Human prostate organoids were a kind gift from Yu Chen (Memorial Sloan Kettering Cancer Center, New York) [19]. They were maintained in complete AdDMEM/F12 supplemented with 50 × diluted B27 (Life Technologies), 10 mM Nicotinamide (Sigma-Aldrich), 1.25 mM N-acetyl-L-cysteine (Sigma-Aldrich), 10 μM SB202190 (Sigma-Aldrich), 5 ng/mL EGF (PeproTech), 5 ng/mL FGF2 (PeproTech), 10 ng/mL FGF10 (PeproTech), 500 nM A83-01 (Tocris Bioscience), 1 μM prostaglandin E2 (Tocris Bioscience), 1% Noggin conditioned medium, 10% R-spondin 1 conditioned medium, 1 nM DHT (Sigma-Aldrich), and 10 μM Y-27632 dihydrochloride (PeproTech). Culture medium was replenished every 3–5 days.

### Compounds and inhibitors

**Fig. 1:**
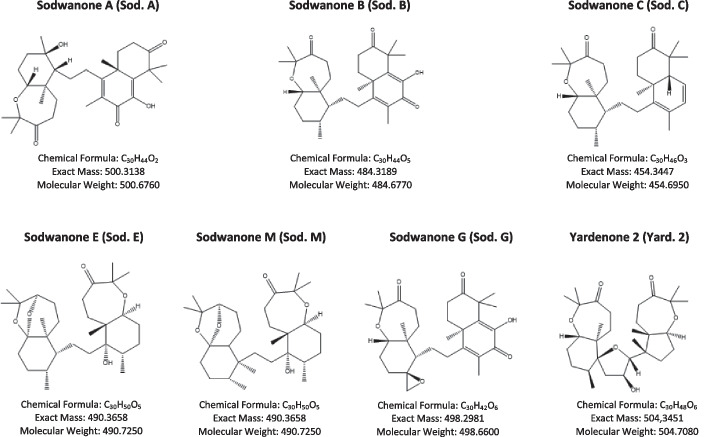
Structures of Sodwanones A-C (1–3), G (4), and Yardenone 2 (5) from Pr. M. Mehiri (Institute of Chemistry of Nice (ICN))

- HIF-1 inhibitors: Sodwanones and Yardenones from Pr M. Mehiri **(**Institute of Chemistry of Nice (ICN)) (Fig. 1 and Table 1).
- shRNA

**Table 1:** The mass and solvent of all the compounds

Compound	Mass	Solvent
Sodwanone A (Sod. A)	17.6 mg	DMSO
Sodwanone B (Sod. B)	2.3 mg	DMSO
Sodwanone C (Sod. C)	6.4 mg	DMSO
Sodwanone E/M (Sod. E/M)	11.4 mg	DMSO
Sodwanone G (Sod. G)	1.1 mg	DMSO
Yardenone 2 (Yard. 2)	9.7 mg	DMSO

Doxycycline-inducible shRNA expression vectors (Addgene) targeting HIF1α (pLKO.1-TetON-Puro-HIF-1α 0819) were engineered by cloning oligonucleotides containing the targeting sequences identical to TRCN0000010819 (TGCTCTTTGTGGTTGGATCTA) into the *Age*I/*EcoR*I sites of a pLKO-TetOn-Puromycin shRNA vector [20]. A non-targeting doxycycline-inducible shRNA (pLKO-Tet-On-shRNA-Control) was used as a control (target sequence: CAACAAGATGAAGAGCACCAA).

### Proliferation

P69, DU145, PC3 and 786-O cells were cultured in 6-well plates for 48 h in the absence (0 μM—DMSO as control), or presence of potential HIF-1 inhibitors at 10 μM, 20 μM and 30 μM. Cells were exposed to normoxia (21% O_2_) or hypoxia (1% O_2_). Cell counting was done using ADAM device on two independent dishes at each time point.

### Clonogenicity

10,000 cells were seeded onto 6 well-plates with 5 mL medium, in the absence or presence of potential HIF-1 inhibitors (10 μM or 20 μM) under normoxic or hypoxic (1% O_2_) conditions 7d or 10d. Medium was removed and 1 mL PFA (20 min) was added to fix the cells. Dishes were then stained with 1 mL crystal violet (0.4%) for 15 min and then rinsed with PBS.

### Cell cycle analysis by flow cytometry

PC3 cells were cultured at a concentration of 200,000 cells/well in 6-well plates in hypoxia (1% O_2_) for 48 h with or without the treatment (20 µM Yard. 2 or 30 nM DTX). Cells were collected by trypsinization and fixed in 70% (v/v) ethanol at 4 °C for at least 4 h. The fixed cells were rinsed twice with PBS and resuspended in 50 µl PBS containing 100 mg RNAse (Invitrogen) for an incubation of 5 min at 4 °C. Subsequently, 450 μl FACS buffer (2% BSA in PBS) containing 25 μg PI was added. After incubation at 4 °C for 30 min, analysis was performed immediately on MACSQuant® Analyzer 10 Flow Cytometer (Miltenyi Biotec).

### RNA-seq

PC3 cells were cultured in 6-well plates in normoxia for 24 h as a control. In parallel, PC3 cells were cultured in hypoxia (1% O_2_) for 48 h in the absence or presence of Yard. 2 (20 μM) or DTX (30 nM). Growth medium was discarded, and cells detached in 1X PBS with a rubber policeman. Resuspended cells were collected in PBS and centrifuged at 1500 rpm for 5 min, then the supernatant was discarded, and the cell pellet was collected. The sample was immediately frozen in − 80℃ and shipped to BGI (Hong Kong) along with adequate dry ice to be analyzed. The data were analyzed using different platforms. Venn diagram for differentially expressed genes was performed on Bioinformatics and Evolutionary Genomics (Draw Venn Diagram (ugent.be)). Phantasus v1.11.0 (https://artyomovlab.wustl.edu/phantasus) was used for drawing heatmap and performing principal component analysis, ShinyGo 0.76 (ShinyGO 0.76 (sdstate.edu)) was used for molecular component and molecular function analysis.

### Immunofluorescence

Cells were fixed in 4% paraformaldehyde (PFA) and permeabilized with 0.2% Triton X-100. Primary antibodies included HIF-1α polyclonal antibody (Invitrogen, ThermoFisher scientific; 1:400 dilution); anti-acetylated tubulin (Sigma-Aldrich, Basel, Switzerland; 1:400 dilution). Alexa Fluor 594- and 488-conjugated secondary goat anti-mouse or goat anti-rabbit antibodies (Molecular Probes, Carlsbad, CA) were used at 1:200. DAPI was used at 1:100. Cells were visualized by wide-field, confocal fluorescence microscopy using a DM5500B upright stand (Leica, Germany) with a 63X oil objective NA 1.00. The cubes used were A4 (excitation filter BP 360/40, dichroic mirror 400, emission filter BP 470/40), L5 (BP 480/40, 505, BP 527/30) and TX2 (BP 560/40, 595, BP645/75). Acquisitions were done with an Orca-ER camera (Hamamatsu, Japan). The microscope was equipped with an automated xy stage. HIF-1α frequency was counted manually from scans using ImageJ for 100–300 nuclei.

### Immunoblotting

Cells were lysed in 1.5 × Laemmli buffer and the protein concentration determined using the BCA assay. 40 µg of protein from whole cell extracts was resolved by SDS-PAGE and transferred onto a PVDF membrane (Millipore). Membranes were blocked in 5% non-fat milk in TN buffer (50 mM Tris–HCl pH 7.4, 150 mM NaCl) for 1 h and incubated in the presence of the primary antibodies in 5% non-fat milk in TN buffer overnight. Then the membranes were incubated with secondary antibodies in 5% non-fat milk in TN buffer. After washing in TN buffer containing 1% Triton-X100 and then in TN buffer, immunoreactive bands were visualized with the ECL system (Amersham Biosciences). Mouse polyclonal anti-HIF-1α antibody (610959) was purchased from *BD Bioscience*. Mouse anti-α-tubulin antibody (MA5-16308) was from *Sigma* and mouse anti-β-actin antibody (MA5-15739) from Invitrogen. Rabbit anti-HIF-2α antibody (NB100-122) was from *Novus Biologicals*. ECL signals were normalized to α-tubulin or -β-actin.

### Statistics

All values are the means ± SEM. Statistical analyses were performed using paired t-test, the ordinary one-way ANOVA and 2-way ANOVA tests in Prism. The *p* values are indicated. All categorical data used numbers and percentages. Quantitative data were presented using the median and range or mean. All statistical tests were two-sided, and *p*-values < 0.05 indicated statistical significance while *p*-values between 0.05 and 0.10 indicated a statistical tendency.

## Results

### Yardenone 2 destabilizes HIF-1α at the protein level, reducing its ability to translocate into the nucleus

**Fig. 2:**
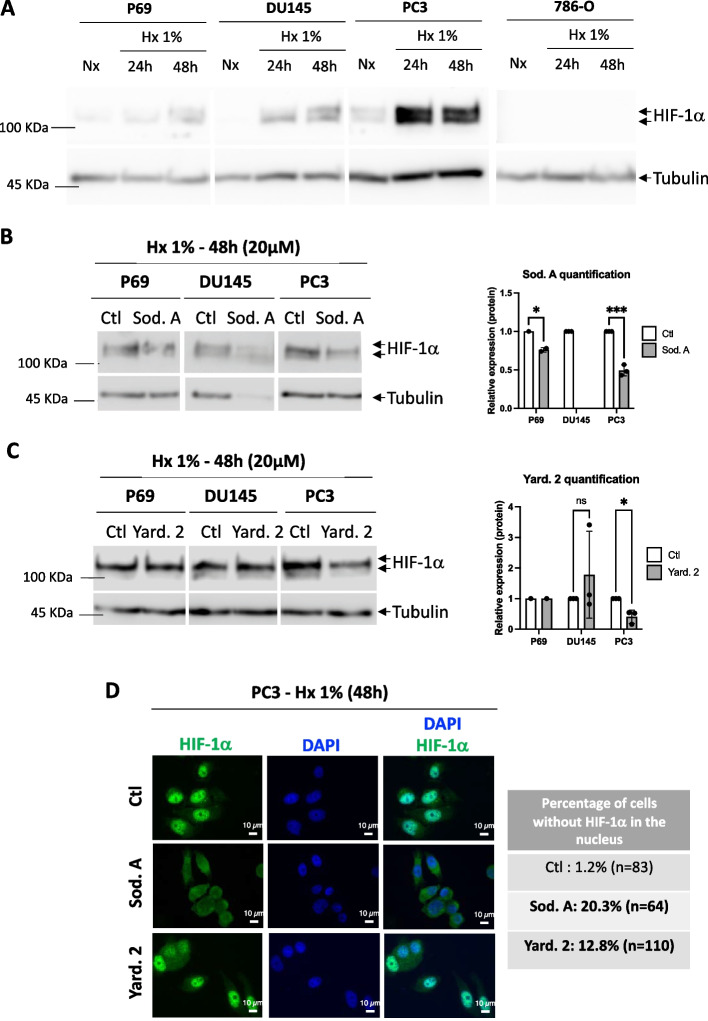
Effect of Sodwanones and Yardenones on HIF-1α stabilization. **A** P69, DU145, PC3, and 786-O cells were subjected to hypoxia 1% O_2_ (Hx 1%) for 24 and 48h. Cell lysates were analyzed by immunoblotting for HIF-1α. Tubulin was used as a loading control. **B** P69, DU145, and PC3 cells were treated with Sodwanone A (Sod. A) at 20 μM for 48h in hypoxia (Hx 1%). Cell lysates were analyzed by immunoblotting for HIF-1α. Tubulin was used as a loading control. **C**, P69, DU145, and PC3 cells were treated with Yardenone 2 (Yard. 2) at 20 μM for 48h in hypoxia (Hx 1%). Cell lysates were analyzed by immunoblotting for HIF-1α. Tubulin was used as a loading control. **D,** Immunofluorescence labeling and merge images showing the nuclear localization of HIF-1α (in green) and DAPI (in blue) in PC3 cells treated with Sod. A and Yard. 2 at 20 μM for 48h in hypoxia (Hx 1%)

We initially evaluated the presence of HIF-1α by immunoblot in the various cells, including P69 normal epithelial cells, DU145 and PC3 prostate cancer cells and 786-O kidney cancer cells. The 786-O cells served as a negative control due to their exclusive expression of HIF-2. We exposed the cells to 1% O_2_ hypoxia (Hx) for 24- and 48-h and used immunoblotting to assess the presence of HIF-1α. Our results confirmed the absence HIF-1α of in the 786-O cells (Fig. 2A). We conducted an initial screening for the destabilization of HIF-1α under hypoxic conditions using Sodwanone A, B, C, E/M, G and Yardenone 2 (Fig. 1). At 10 µM, no significant effects were observed, while 30 µM was excessively toxic, resulting in a 45% decrease in cell viability in P69 cells, an 89% decrease in DU145 cells, and a 48% decrease in PC3 cells (unpublished data). We thus selected 20 µM as the optimal concentration. Sod. A effectively destabilized HIF-1α in P69 and PC3 cells but remained toxic to DU145 cells (Fig. 2B). Interestingly, Yard. 2 exclusively destabilized HIF-1α in PC3 cells (Fig. 2C). Subsequently, we focused our investigation on Sod. A and Yard. 2. To investigate the impact of these compounds on HIF-1α localization for dimer formation and subsequent gene activation, we performed immunofluorescence analyses. In PC3 cells exposed to 48 h of hypoxia, Sod. A reduced HIF-1α nuclear localization by 20.3%, while Yard. 2 significantly decreased nuclear HIF-1α, reduced to 12.8% (Fig. 2D).

We also investigated a possible effect at the level of transcriptional regulation (Suppl. Figure 1). While the mRNA expression level of HIF-1α was decreased under hypoxia in conditions where the HIF-1α protein is strongly stabilized, we did not observe any effect of Yard. 2 on the mRNA expression level of HIF-1α in PC3 cells. This indicates that the destabilization of HIF-1α by Yard.2 occurs at the protein level, rather than at the transcriptional level.

These findings confirm the targeted action of both Sod. A and Yard. 2. Sod. A exhibits significant toxicity in hypoxic DU145 cells, while Yard. 2 selectively affects PC3 cells, which are known to be among the most aggressive PCa cell lines.

### Yardenone 2 selectively reduces the proliferation of PC3 cells only under hypoxic conditions

**Fig. 3:**
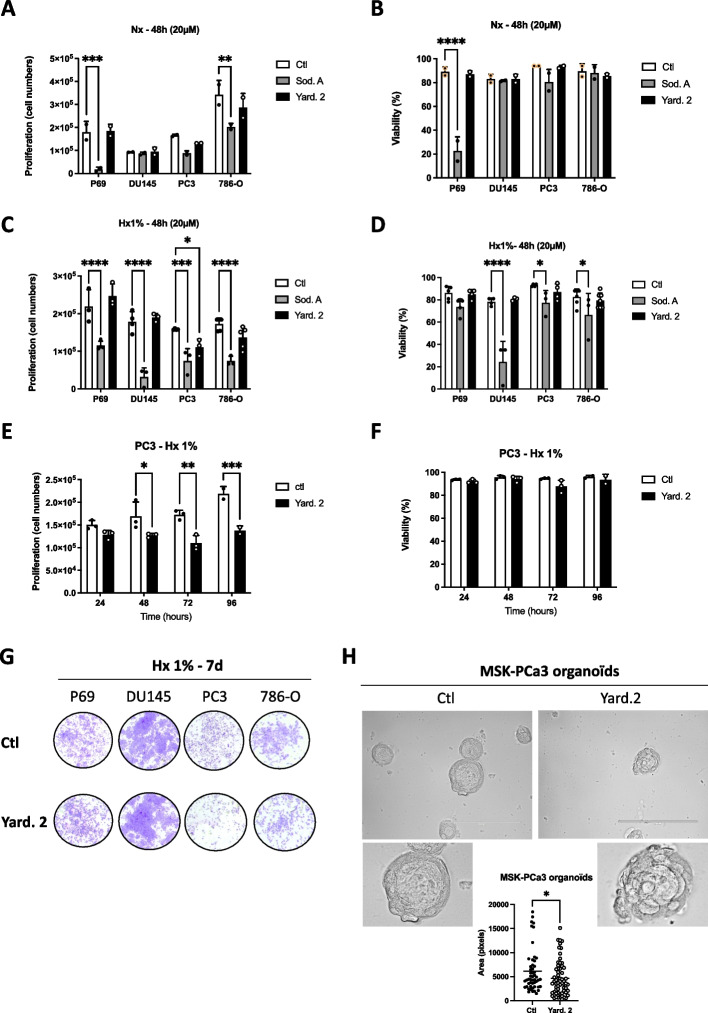
Impact of the Sodwanone A (Sod. A) and Yardenone 2 (Yard. 2) on cell proliferation and viability.** A** and **B** P69, DU145, PC3, and 786-O cells were seeded at the same density and incubated in normoxia (Nx) for 48 h in the absence (Ctl) or presence of 20 µM of Sod. A, and Yard. 2. Cell proliferation (**A**) and cell viability (**B**) were measured using an ADAM cell counter. **C** and **D** P69, DU145, PC3, and 786-O cells were seeded at the same density and incubated in hypoxia (Hx 1%) for 48 h in the absence (Ctl) or presence of 20 µM of Sod. A, and Yard. 2. Cell proliferation (**C**) and cell viability (**D**) were measured using an ADAM cell counter. (**E** and **F**) PC3 cells were seeded at the same density and incubated in hypoxia (Hx 1%) for 24, 48, 72, and 96 h in the absence (Ctl) or presence of 20 µM of Yard. 2. Cell proliferation (**E**) and cell viability (**F**) were measured using an ADAM cell counter. **G**, Clonogenic assay of P69, DU145, PC3, and 786-O cells. Cell lines were seeded at the same density and incubated in Hx 1% O_2_ (Hx 1%) for 7 days (7d) in the absence (Ctl) or presence of Yard. 2 at 20 µM. **H** Top, Three-dimensional structures obtained from confocal image series using IMARIS software; scale bars = 200 µm. MSK-PC3 organoids have been treated for 15 days in the absence or presence of Yard. 2 (40 µM) every 3 days. Bottom, Quantification of cell area (pixels) at day 15. The 2-way ANOVA is representative of at least five different organoids. *p < 0.05, **p < 0.01, ***p < 0.001, ****p < 0.0005. Data represent the mean ± SD of experiments performed at least three times

We then examined the effect of Sod. A (20 µM) or Yard.2 (20 µM) on the proliferation and viability of P69, DU145, PC3 and 786-O cells. In normoxia (Nx), 20 µM Sod. A significantly decrease proliferation at 48 h in both P69 and 786-O cells and reduced cell viability (Fig. 3A and B). In contrast, Sod.A had no effect on the proliferation or viability of DU145 and PC3 cells. Similarly, Yard. 2 did not impact any of the cell lines tested (Fig. 3A and B).

We validated the effects of the drug responses under hypoxic conditions. Sod. A displayed a significant negative impact on all cell lines tested, including 786-O cells that do not express HIF-1 (Fig. 3C). Viability was reduced in DU145, PC3 and 786-O cells (Fig. 3D). In contrast, Yard. 2 specifically decreased proliferation in PC3 cells without causing cell death (Fig. 3C and D). Consequently, we chose to focus exclusively on Yard. 2, which appears to be more selective under hypoxic conditions and with respect to HIF-1. Over an extended period of up to 96 h, Yard. 2 significantly inhibits proliferation under hypoxic conditions, without affecting PC3 cell viability (Fig. 3E and F). Following a 48-h incubation with 20 µM of Yard. 2 and negative controls, cell cycle stages were analyzed using FACS (Suppl. Figure 2). The results demonstrated a significant accumulation of cells in the G2/M phase (3.5 ± 0.78% for Yard. 2). However, Yard. 2 did not influence the cell cycle in 786-O cells, suggesting that this effect could be dependent on HIF-1. These findings indicate that Yard. 2 may slightly inhibit proliferation by arresting the cell cycle at the G2/M phase. Clonogenic assays, commonly used in cancer research to assess the ability of cancer cells to initiate tumors, revealed that Yard. 2 significantly reduces clonogenicity without inducing cell death over a 7-day period, as evidenced by the lack of colony growth in PC3 cells (Fig. 3G). To further validate Yard. 2's effectiveness in a 3D model, we utilized patient-derived organoid lines, specifically MSK-PCa3, from Chen’s laboratory, which mimic the molecular diversity of adenocarcinoma prostate cancer. These organoids were cultured for 15 days with Yard. 2 being added every 3 days (Fig. 3H). In the presence of Yard. 2, we observed a significant decrease in the size of the organoids, along with notable changes in their morphology and conformation. Together, these results strongly suggest that, Yard. 2 may serve as a specific HIF-1 inhibitor, acting at the protein level with a cytostatic effect. In contrast, Sod. A has unfavorable effects in both normoxia or hypoxia, regardless of HIF-1 status or whether the cells are tumoral or normal.

### Yardenone 2 specifically acts on HIF-1α target genes

To validate the effects of Yard. 2 on HIF-1 and gene expressions, we performed an RNA-seq experiment using PC3 cells in both normoxia and hypoxia, in the absence or presence of Yard. 2 for 48 h.

**Fig. 4:**
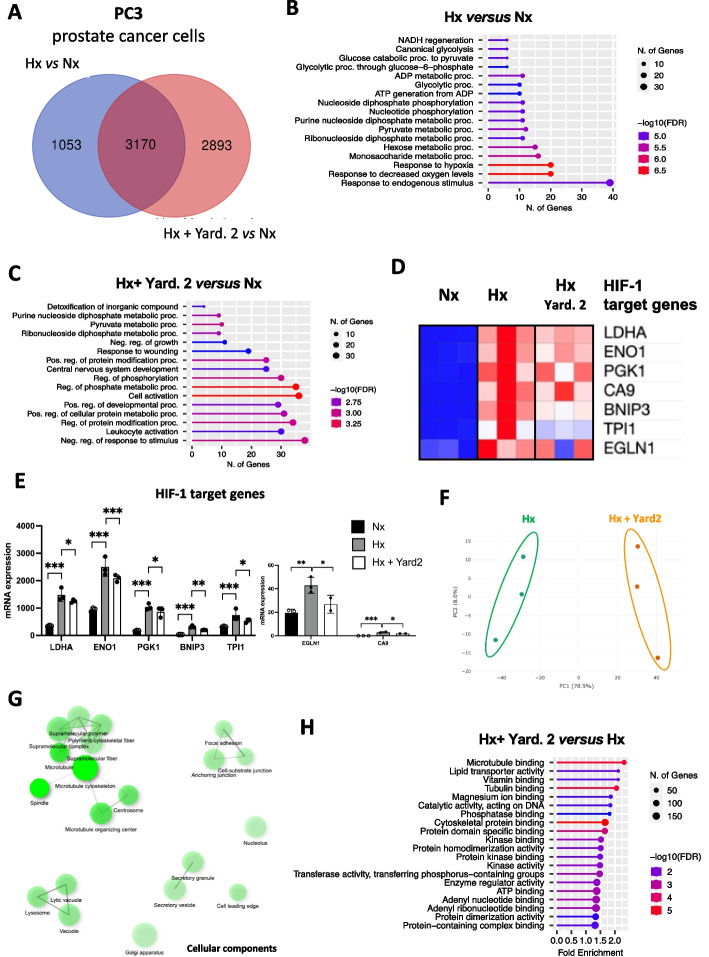
Yard. 2 modulates HIF-1 target genes and acts as a microtubule inhibitor. PC3 cells were treated in normoxia (Nx) and hypoxia (Hx 1%) in the absence or presence of Yard. 2 at 20μM for 48h. **A** Venn diagram showing the differential distribution of the genes detected between hypoxia and normoxia (in blue) and between Hx + Yard. 2 and Nx (in red). **B** Gene set enrichment list of RNA-Seq data comparing hypoxia (Hx) and normoxia (Nx) using “Cellular functions”. **C** Gene set enrichment list of RNA-Seq data comparing hypoxia + Yard. 2 (Hx + Yard. 2) and normoxia (Nx) using “Cellular functions”. **D** and **E** Heatmap (**D**) and the representative bar diagram (**E**) of some specific HIF-1 target genes in normoxia (Nx) compared to hypoxia (Hx) or hypoxia treated with Yard. 2. **F** PCA plot of hypoxia (Hx) and hypoxia + Yard.2 (Hx + Yard. 2) samples. Ellipses and shapes show clustering of the samples. **G** gene set enrichment map of RNA-Seq data comparing hypoxia + Yard. 2 (Hx + Yard. 2) and hypoxia (Hx) using “Cellular components”. **H** Gene set enrichment list of RNA-Seq data comparing hypoxia + Yard. 2 (Hx + Yard. 2) and hypoxia (Hx) using “Molecular functions”

The analysis identified 4,223 differentially expressed genes between Hx and Nx, with a significance cut-off of *p*-values < 0.05. Furthermore, in hypoxic cells treated with Yard. 2, 6063 genes exhibited differential expression compared to Nx, with the same *p*-value threshold. Notably, the expression of 2893 genes was significantly altered in PC3 cells upon Yard. 2 treatments (Fig. 4A). Analysis of the top molecular functions between hypoxia and normoxia revealed significant changes in pathways, including NADH regeneration, glycolytic process, and notably, an emphasis on the response to hypoxia and low oxygen levels (Fig. 4B). Interestingly, the addition of Yard. 2 under hypoxic conditions compared to normoxic conditions resulted in the elimination of the hypoxic response, highlighting the potential effectiveness of Yard. 2 in hypoxia (Hx) (Fig. 4C). We then examined the modulation of HIF-1 target genes to determine whether Yard. 2 had a specific impact in Hx. Significant differences were observed in the expression of HIF-1 target genes, including *Ldha*, *Eno1*, *Pgk1*, *Ca9*, *Bnip3*, *Tpi1* and *Egln1* (Fig. 4D and E). In contrast, no significant changes were found in the expression of HIF-2 target genes, such as *Hes4*, *Cdt1* and *Pparg* (Suppl. Figure 3A and B), suggesting a selective effect of Yard. 2 on HIF-1 target genes. Additionally, we identified 11 distinct clusters of genes with varying expression patterns when comparing hypoxia to normoxia (Suppl. Figure 3C). Principal component analysis (PCA) further confirmed the difference between Hx and Nx, indicating homogeneous samples, particularly in Nx, and distinct separation between the two groups (Suppl. Figure 3D). Analysis of cellular components revealed enrichment in mitochondria and supramolecular fibers (Suppl. Figure 3E). When PC3 cells were treated with Yard. 2, we identified 11 clusters of differentially expressed gene with distinct functions (Suppl. Figure 3F). The PCA showed samples homogeneity in both groups, with a clear separation between them (Fig. 4F). In PC3 cells treated with Yard. 2, an enrichment of cellular components such as microtubule, cytoskeleton, focal adhesion, lysosome, secretory vesicle was observed (Fig. 4G). The molecular functions characterized were consistent with the observed cellular components, including microtubule binding, tubulin binding, cytoskeletal protein binding, ATP binding and protein dimerization activity (Fig. 4H).

These findings provide strong evidence of the modulation of HIF-1 target genes in the presence of Yard. 2, highlighting its influence on various processes occurring during hypoxic culture conditions. Moreover, Yard.2 appears to have a significant impact on microtubule-related processes.

### Docetaxel and Yard. 2 exhibit similarities and distinctions in their actions on microtubules in hypoxia

To gain deeper insights into the mode of action of Yard. 2 on microtubules, we used Docetaxel (DTX) as a positive control. DTX selectively binds to microtubules, which are essential for cell division, thereby disrupting cell division. This disruption prevents the proper separation of chromosomes during cell division, resulting in the arrest of cancer cell growth. In the context of prostate cancer, DTX is commonly used as first-line chemotherapy for patients with castration-resistant prostate cancer (CRPC) [21, 22].

**Fig. 5:**
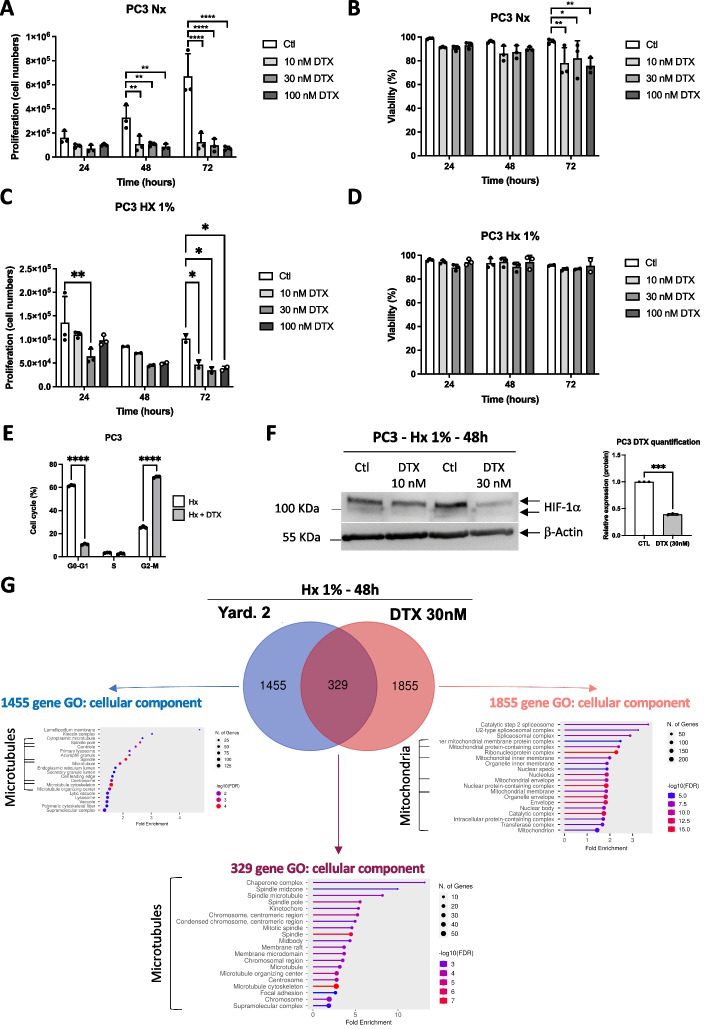
Docetaxel (DTX) and Yard. 2 exhibit similar but distinct actions. **A** and **B** PC3 cells were incubated in normoxia (Nx) for 24, 48h, and 72h in the absence (Ctl) or presence of 10, 30 or 100nM of Docetaxel (DTX). Cell proliferation (**A**) and cell viability (**B**) were measured using an ADAM cell counter. **C** and **D** PC3 cells were incubated in hypoxia (Hx 1%) for 24, 48h, and 72h in the absence (Ctl) or presence of 10, 30 or 100nM of Docetaxel (DTX). Cell proliferation (**C**) and cell viability (**D**) were measured using an ADAM cell counter. **A**–**D** * *p* < 0.05, ** *p* < 0.01, *** *p* < 0.001, **** *p* < 0.0005. Data represent the mean ± SD of experiments performed at least three times. **E** Cell cycle effect of PC3 cells treated with 30nM of DTX. **F** PC3 cells were treated with 10 or 30nM of Docetaxel (DTX) and subjected to hypoxia 1% O_2_ (Hx 1%) for 48h. Cell lysates were analyzed by immunoblotting for HIF-1α. β-Actin was used as a loading control. **G** PC3 cells were treated in hypoxia (Hx 1%) in the presence of Yard. 2 at 20 μM and in the presence of Docetaxel (DTX) at 30nM for 48h. Venn diagram showing the differential distribution of the genes detected between Hx + Yard. 2 and Hx (in blue) and between Hx + DTX and Hx (in red). Gene set enrichment list of RNA-Seq data using “Cellular functions”

Consequently, we investigated the impact of varying concentrations of DTX (10, 30, and 100 nM) on the proliferation and viability of PC3 cells. Notably, even at the lowest concentration of 10 nM, a noticeable reduction in the proliferation was observed, consistent with the effects observed at 30 and 100 nM (Fig. 5A). Moreover, all concentrations led to a decrease in the viability of normoxic PC3 cells following 72 h of treatment (Fig. 5B). Subsequently, we examined the effects of DTX under hypoxic conditions. We observed a trend of notable negative impact at 24 and48h, while at 72 h, DTX demonstrated a significantly strong negative effect across all tested concentrations (Fig. 5C). Interestingly, under hypoxic conditions, viability was no longer affected (Fig. 5D). After a 48-h incubation with 30 nM of DTX and negative controls, cell cycle stages were analyzed using FACS (Fig. 5E). The results revealed a significant and strong accumulation of cells in the G2/M phase, with 69.13 ± 0.72% for DTX compared to Yard. 2 (Suppl. Figure 2). These findings suggest that inhibition of proliferation due to DTX is likely triggered by cell cycle arrest at the G2/M phase. We then examined the responses to DTX on HIF-1α stabilization, considering prior findings by several groups such as Escuin et al*.* [23], Carbonaro et al*.* [24] and Li et al*.* [25]. These studies reported the destabilization of HIF and an augmentation in cell death following DTX treatment. Although 10 nM was ineffective at 48 h post-treatment, 30 nM notably destabilized HIF-1α to an extent of 60.6 ± 0.4% (Fig. 5F), a more significant effect than observed in the presence of Yard. 2 (Fig. 2C).

To assess the impact of DTX on gene expression patterns, we performed an RNA-seq experiment using PC3 cells in hypoxia with 30 nM of DTX for 48 h. We investigated the modulation of HIF-1 genes target genes to determine any specific impact of DTX. Despite the destabilization of HIF-1α by DTX at 30 nM, we observed either significant up-regulation or no noticeable changes in the expression levels of HIF-1 target genes, including *LDHA*, *ENO1*, *PGK1*, *CA9*, *BNIP3*, *TPI1* and *EGLN1* (Suppl. Figure 4A and B). Moreover, no significant differences were detected in the expression of HIF-2 target genes, such as *HES4*, *CDT1* and *PPARG* (Suppl. Figure 4C and D). Further analysis revealed that 329 genes exhibited common expression patterns when treated with Yard. 2 or DTX, with a significance cut-off of *p*-values < 0.05 (Fig. 5G). These genes were specifically associated with cell cycle, mitotic nuclear division, spindle organization and strongly linked to microtubules. Additionally, 1,855 genes displayed differential expression in response to DTX compared to Yard. 2, also with *p*-values < 0.05, primarily with mitochondrial functions. Notably, the expression of 1,455 genes exhibited significant changes upon treatment with Yard. 2 in PC3 cells compared to DTX, predominantly associated with microtubules, thereby underlying the role of Yard. 2.

These findings suggest that both Yard. 2 and DTX have comparable effects on microtubules while manifesting distinct specificities. Intriguingly, although both compounds affect HIF-1α stabilization, Yard. 2 influences HIF-1 target genes, while DTX does not, pointing to potential differences in their kinetic mechanisms of action.

### Invalidation of HIF-1α affect slightly the microtubule structure

**Fig. 6:**
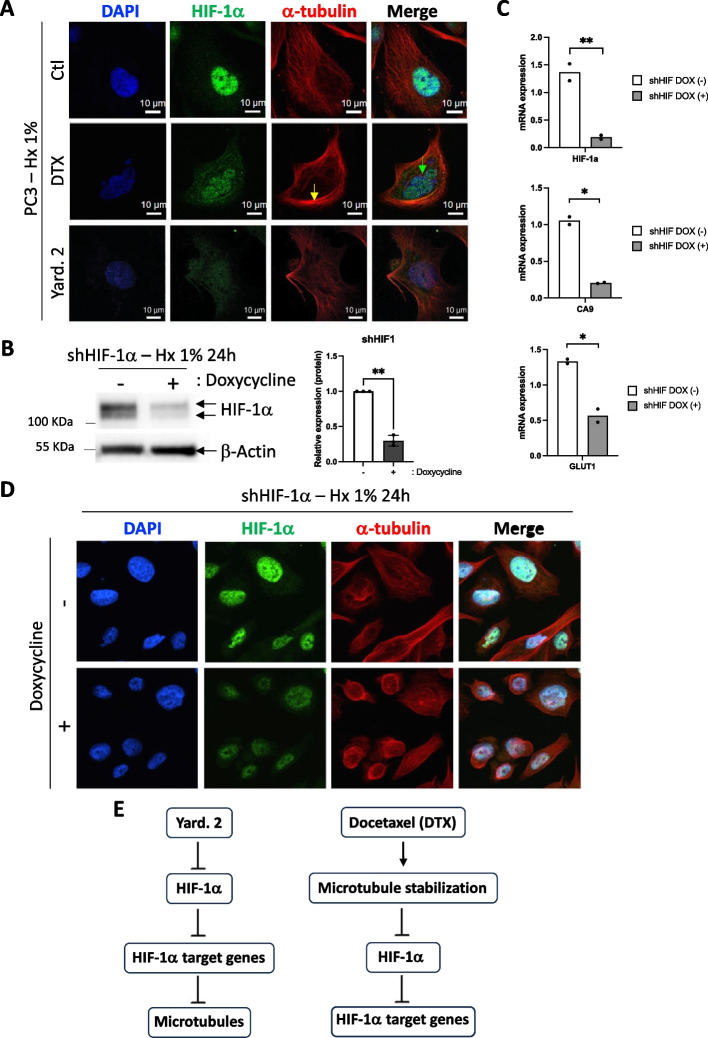
Yard. 2 directly targets HIF-1α, subsequently inhibiting the induction of genes that later impact tubulin. **A** Immunofluorescence labeling and merged images with HIF-1α (in green), α-tubulin (in red), and DAPI (in blue) for PC3 cells treated with 30 nM of Docetaxel (DTX) or 20 µM of Yard. 2 and incubated for 48 h in Hx. The yellow arrow indicates condensed tubulin, while the green arrow signifies mitotic catastrophe. **B** PC3 cells were stably transfected with the inducible (doxycycline) pLKO-TetOn-Puromycin encoding shRNA to HIF-1α. PC3 expressing shRNA HIF-1α were subjected to hypoxia (Hx1%) in the absence (−) or presence ( +) of doxycycline. Cell lysates were analyzed by immunoblotting for HIF-1α. β-Actin was used as a loading control. **C** Graphic representation of *HIF-1α*, *Ca9* and *Glut1* mRNA expression in PC3 cells incubated in hypoxia (Hx—1% O_2_) %) in the absence (−) or presence ( +) of doxycycline for 48 h. The one-way ANOVA is representative of two independent experiments. **D** Immunofluorescence labeling and merged images with HIF-1α (in green), α-tubulin (in red), and DAPI (in blue) for PC3 expressing shRNA HIF-1α in the absence (−) or in the presence ( +) of doxycycline and incubated for 48 h in Hx. **E** Graph illustrating the comparison of the modes of action of Yard. 2 versus Docetaxel

Prolonged mitotic arrest induced by DTX triggers mitotic catastrophe. During this process, cells experience aberrant mitotic events, such as chromosome misalignment, chromosome breakage, and multipolar spindle formation. These abnormalities result in the activation of cell death pathways, leading to apoptosis or necrosis. Consequently, we investigated whether Yard. 2 could provoke similar effects. Immunofluorescence analysis revealed evidence of mitotic catastrophe induced by DTX, even in hypoxia, as indicated by condensed tubulin and the entrapment of nuclei (Fig. 6A). In contrast, treatment with Yard. 2 led to a slight condensation of tubulin, with no instances of multiple nuclei per cell. Given that the level of destabilization of HIF-1α was lower with Yard. 2 compared to DTX, we chose to silence HIF-1α using a doxycycline-inducible shHIF-1α. Following a 24-h treatment, HIF-1α was destabilized to 70% (Fig. 6B) decreasing the mRNA expression levels of *HIF-1α*, *CA9* and *GLUT1* by 86%, 80% and 54%, respectively (Fig. 6C). Given that the level of HIF-1α destabilization was comparable to that induced by Docetaxel, we further investigated whether this effect was associated with mitotic catastrophe. Following a 24-h treatment, tubulin condensation was observed, similar to the effects seen with Yard. 2. However, no evidence of mitotic catastrophe was noted (Fig. 6D).

These results suggest that the direct action on HIF-1α via Yard. 2 or shHIF-1α is the initial event triggering a minor effect on microtubules, whereas DTX treatment likely targets microtubules first, leading to destabilization of HIF-1α (Fig. 6E).

## Discussion

Our study reveals, for the first time, the impact of the marine compound Yardenone 2 (Yard. 2) on the HIF-1α protein in prostate cancer cells. Through our investigations, we have demonstrated that, Yard. 2 has several noteworthy effects among various Sodwanones and Yardenones tested: (1) destabilization of the HIF-1α protein, (2) reduction in the nuclear localization of HIF-1α, and (3) consequent inhibition of PC3 cell proliferation under hypoxic conditions. Furthermore, we demonstrated that, Yard. 2 possesses the ability to modulate the expression of numerous genes (1455 genes) and diverse processes, all of which are implicated in tumor development.

Although Dai et al*.* demonstrated the action of Sodwanones and Yardenones on breast and prostate cancer, our screenings and conclusions differed [16]. They reported that Sodwanone V inhibited hypoxia- and iron chelator (1,10-phenanthroline)-induced HIF-1 activation in T47D breast tumor cells (IC50 15 µM). Additionally, Sodwanone V was the sole Sodwanone that inhibited HIF-1 activation in PC3 prostate tumor cells (IC50 15 µM). Other compounds like Sod. K, Sod. T, 10,11-dihydroSodwanone B, and Sod. A inhibited hypoxia-induced activation of HIF-1 in T47D cells (IC50 values 20-25 µM). 3-epi-Sodwanone K 3-acetate demonstrated cytotoxicity to T47D cells (IC50 22 µM), and Sod. V exhibited cytotoxicity to MDA-MB-231 breast tumor cells (IC50 23 µM). While we did not have access to all the Sodwanones, we shared Sod. A in common. Moreover, we used the same PC3 and DU145 cells and selected similar concentrations for our study. However, our assay methods differed. Dai et al*.* limited their study to reporter gene and luciferase assays, whereas we focused on more physiological processes such as stabilization effects, nuclear entry, and impact on cell proliferation and/or death. Sod. A, initially a focal point of our study, was subsequently characterized for its cytotoxic properties regardless of the presence of HIF-1α or the cell line. Consequently, we chose to eliminate Sod. A from the compounds with therapeutic potential and shifted our focus to Yard. 2. Our findings demonstrate that among the tested Sodwanones and Yardenones, only Yard. 2 destabilized HIF-1α, reduced the nuclear localization of HIF-1α, and inhibited the proliferation of PC3 cells. However, at this point, we cannot determine whether Yard. 2 specifically blocks HIF-1α entry into the nucleus, perhaps by masking NLS sequences [26], or if the simple decrease in HIF-1α results in reduced entry into the nucleus. Only further studies on the kinetics of HIF-1α entry into the nucleus can confirm this hypothesis.

In the presence of Yard. 2, we did not observe significant cell death but rather a cytostatic effect observed over the long term. This effect was evident at 7 days in clonogenicity assays and from 96 h in proliferation readouts. While our study did not yet pinpoint the specific factors responsible for the proliferation blockade, results from FACS analysis indicated a cell cycle arrest in G2/M phase, the specific checkpoint that prevents cells from entering mitosis when DNA is damaged. It is noteworthy that Yard.2 appears to be less aggressive towards cells than DTX, as evidenced by a lower G2/M arrest (3.5% versus 69.13%, respectively). However, the reduction in proliferation observed with Yard. 2 compared to DTX might be attributed to this slight G2/M phase arrest. This G2 arrest is associated with the lack of dephosphorylation of the CycA-CDK1 and CycB-CDK1 complexes. Additionally, CDK1 plays a role in the stabilization of HIF-1α, either through direct phosphorylation of Ser668 [27] or by controlling HSP90 [28]. However, since HIF-1α was destabilized by approximatively 50% in the presence of Yard.2, compared to 60% with DTX, it is unlikely that CDK1 is involved in the destabilization of HIF-1α by Yard. 2.

Cytostatics are substances that block cell synthesis, function, or multiplication. Most of these substances work by arresting mitosis, effectively targeting rapidly dividing cells. The cell cycle is crucial when administering cytostatics, as some agents are most effective when cells are actively dividing, while others work best at specific phases of the cycle, such as G1, S, or G2 [29, 30]. Various cytostatics are available to treat cancer. In most cases, these substances target DNA, RNA, or protein synthesis, which are involved in the formation of the cancer cell's structure. Cytostatics can be classified into various categories based on their mechanisms of action, molecular structures, or sources. These categories include alkylating agents, intercalating agents, antimetabolites, microtubule inhibitors, type I and II topoisomerase inhibitors, and tyrosine kinase inhibitors (https://www.altmeyers.org/en/internal-medicine/cytostatics-classification-141873). While it is not yet clear which category Yard. 2 belongs to, the RNA-Seq results have provided us with some intriguing insights. Yard. 2 appears to be heavily influenced by factors related to microtubules and the cytoskeleton, suggesting that it may belong to the category of microtubule inhibitory agents, similar to Colchicine, Docetaxel, or Vinblastine [31]. However, upon comparing Yard. 2 to DTX, notable differences emerged. Firstly, DTX lacks specificity to hypoxic cells. In fact, cells are even protected in hypoxia, contrary to Yard. 2, which unequivocally demonstrates specificity to hypoxia. Secondly, although both compounds destabilize HIF-1α, they do not affect the HIF-1 target genes in the same manner overtime. Yard. 2 and shHIF-1α decreased the mRNA expression of HIF-1 target genes, while we did not observe such an effect in the presence of DTX. Long-term treatments may be necessary to influence gene expression effectively. Thirdly, unlike DTX, we did not observe mitotic catastrophe with Yard. 2. All these differences raise the question: what makes Yard. 2 so unique or distinctive in its mode of action? The answer lies within the 1455 distinctive genes associated with the kinesin complex, spindle pole, centrosome, and other related factors. From the genes associated with microtubules, we identified 26 genes exhibiting differential expression between Nx and Hx, a discrepancy which was subsequently reversed following Yard. 2 treatments. Among these 26 genes, *Dnal1*, *Map6d1* and *Ndel1* were selected as potential targets to explore their effects in Hx. However, none of these three genes demonstrated a decrease in HIF-1α at either the protein or the mRNA level (data not shown). We are currently exploring other genes, but it is highly unlikely that the silencing of a single gene could be responsible for the destabilization of HIF-1α. It is more probable that a combination of genes exists. The next step involves testing the invalidation of those genes using a siRNA library to characterize the specific genes targeted by Yard. 2 that are truly involved in HIF-1 targeting.

## Conclusion

Our primary challenge lies in the solubility of these compounds. Sodwanones/Yardenones exhibit poor solubility, occasionally resulting in variations in their concentration. Crystallization in DMSO containing Yard. 2 has been observed on occasion. Improving solubility while maintaining their efficacy against HIF-1α remains a significant challenge. Another challenge is the limited quantity of compounds available. While the current supply of Yard. 2 has been sufficient for in vitro experiments, it may not be adequate for in vivo studies. In conclusion, discovering new compounds derived from natural sources with genuine abilities to inhibit HIF-1 or other compounds proves to be highly promising. However, this quest cannot be solely conducted at the biological level. It must be pursued with seamless coordination with cutting-edge chemistry to enable mass production.

### Supplementary Information

Supplementary Material 1. Figure 1. Yard. 2 does not modulates HIF-1 mRNA. PC3 cells were treated in normoxia (Nx) and hypoxia (Hx 1%) in the absence or presence of Yard. 2 at 20 μM for 48h. Representative bar diagram of HIF-1α gene in normoxia (Nx) compared to hypoxia (Hx) or hypoxia treated with Yard. 2.

Supplementary Material 2. Figure 2. A and B, Cell cycle effect of PC3 (A) and 786-O (B) cells treated with 20µM of Yard. 2.

Supplementary Material 3. Figure 3. A and B, Heatmap (A) and the representative bar diagram (B) of some specific HIF-2 target genes in normoxia (Nx) compared to hypoxia (Hx) or hypoxia treated with Yard. 2. C, Heatmap of differentially expressed genes between normoxia (Nx) and hypoxia (Hx). The top molecular functions of the 11 clusters characterized are listed on the right. D, PCA plot of normoxia (Nx) and hypoxia (Hx) samples. Ellipses and shapes show clustering of the samples. E, Gene set enrichment map of RNA-Seq data comparing hypoxia (Hx) to normoxia (Nx) using “Cellular components”. F, Heatmap of differentially expressed genes between hypoxia (Hx) and hypoxia + Yard. 2 (Hx + Yard. 2). The top molecular functions of the 11 clusters characterized are listed on the right.

Supplementary Material 4. Figure 4. A and B, Heatmap (A) and the representative bar diagram (B) of some specific HIF-1 target genes in normoxia (Nx) compared to hypoxia (Hx) or hypoxia treated with Docetaxel (DTX). C and D, Heatmap (C) and the representative bar diagram (D) of some specific HIF-2 target genes in normoxia (Nx) compared to hypoxia (Hx) or hypoxia treated with Docetaxel (DTX).

## Data Availability

Availability of data and materials, all data generated or analyzed during this study are included in this published article.

## Abbreviations

BNIP3: BCL2 interacting protein 3
CA9: Carbonic anhydrase 9
DTX: Docetaxel
EGLN1, also known as PHD2: Egl-9 family hypoxia inducible factor 1
ENO1: Enolase 1
GLUT1: Glucose transporter 1
HIF-1: Hypoxia inducible factor-1
LDHA: Lactate dehydrogenase A
PGK1: Phosphoglycerate kinase 1
Sod.: Sodwanone
TPI1: Triosephosphate isomerase 1
Yard.: Yardenone
