# Supplementary figures and images for: The marine-derived HIF-1α inhibitor, Yardenone 2, reduces prostate cancer cell proliferation by targeting HIF-1 target genes
Source: Cell Mol Biol Lett. 2024 Jul 8;29:101. doi: 10.1186/s11658-024-00617-2 (PMC11232290; doi:10.1186/s11658-024-00617-2)

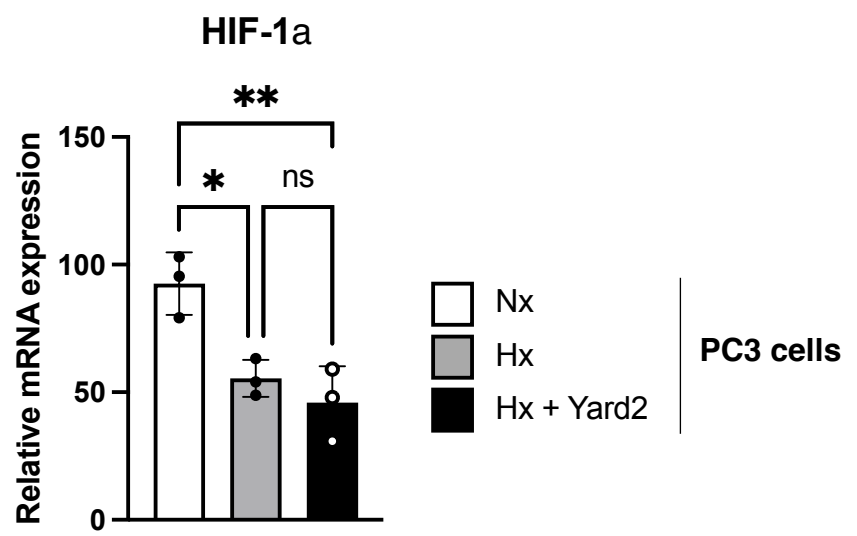

Supplement: Supplementary file 1 — Supplementary Material 1. Figure 1. Yard. 2 does not modulates HIF-1 mRNA. PC3 cells were treated in normoxia (Nx) and hypoxia (Hx 1%) in the absence or presence of Yard. 2 at 20 μM for 48h. Representative bar diagram of HIF-1α gene in normoxia (Nx) compared to hypoxia (Hx) or hypoxia treated with Yard. 2. [file 11658_2024_617_MOESM1_ESM.pdf]

**A****PC3**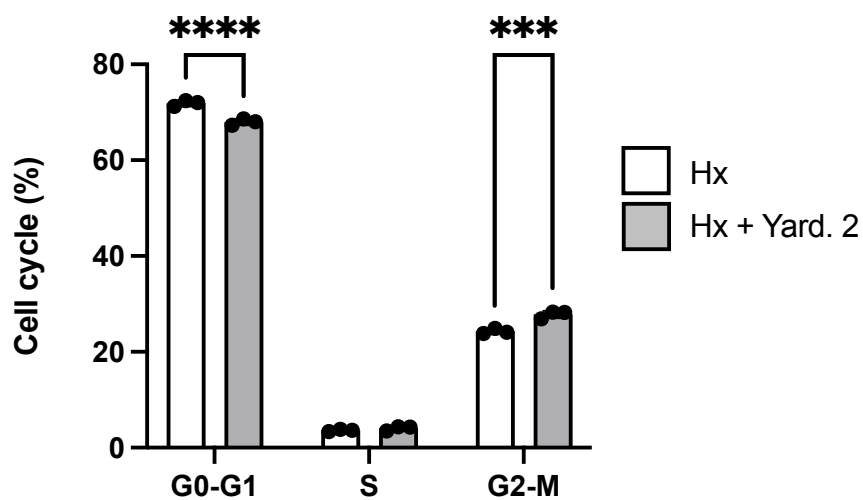**B****786-O**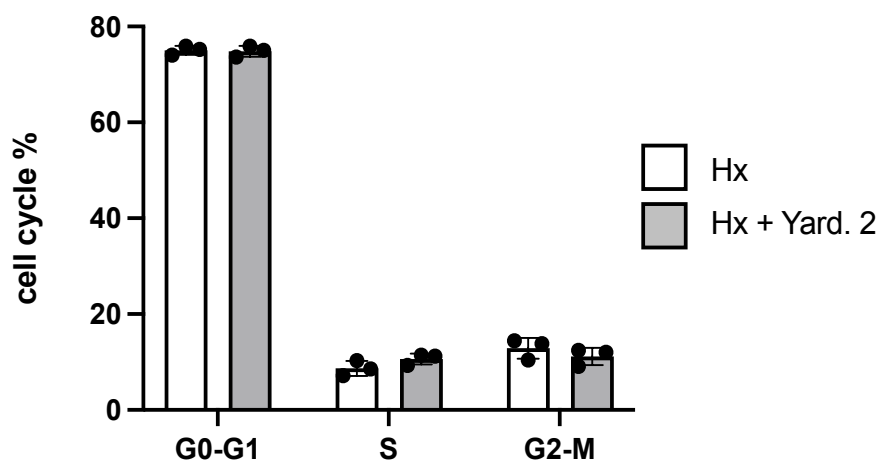

Supplement: Supplementary file 2 — Supplementary Material 2. Figure 2. A and B, Cell cycle effect of PC3 (A) and 786-O (B) cells treated with 20µM of Yard. 2. [file 11658_2024_617_MOESM2_ESM.pdf]

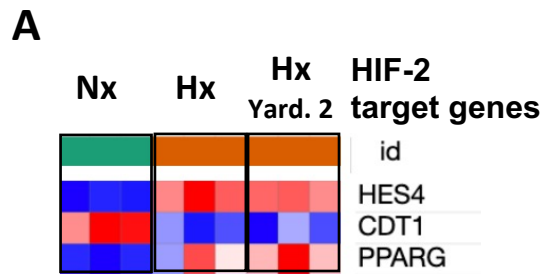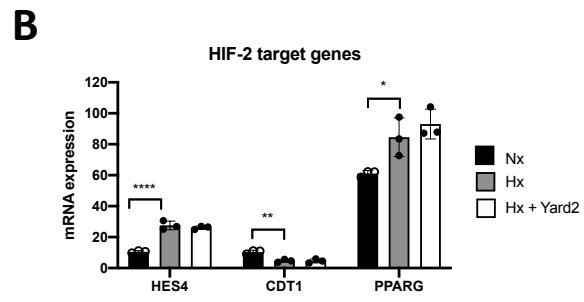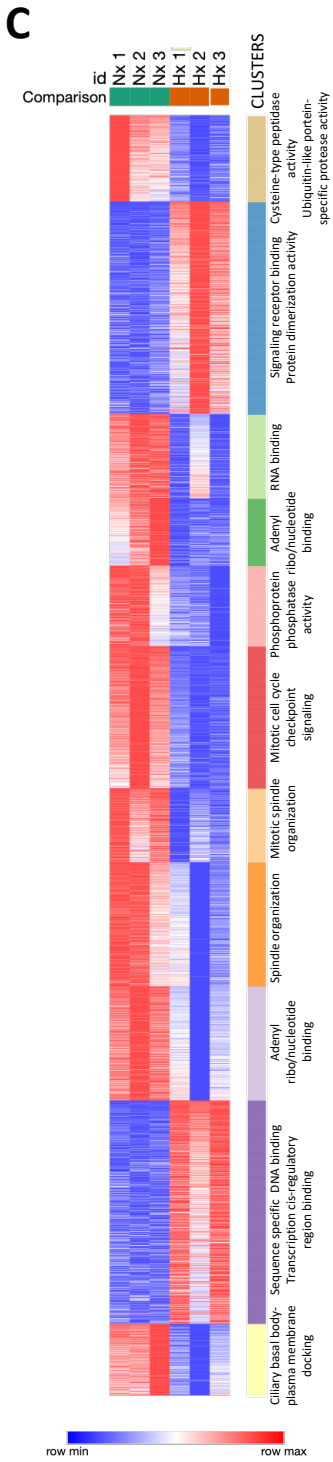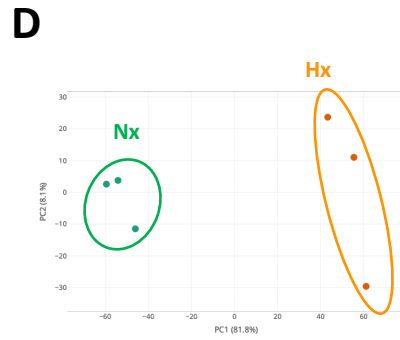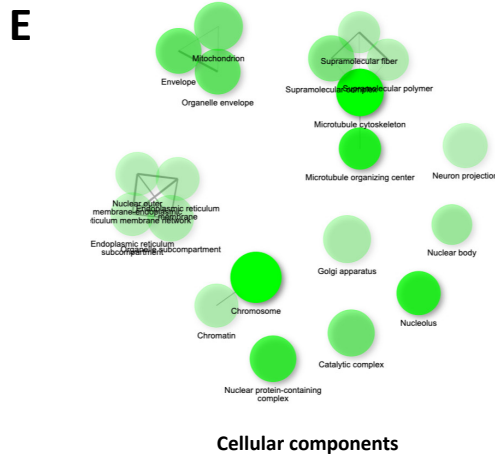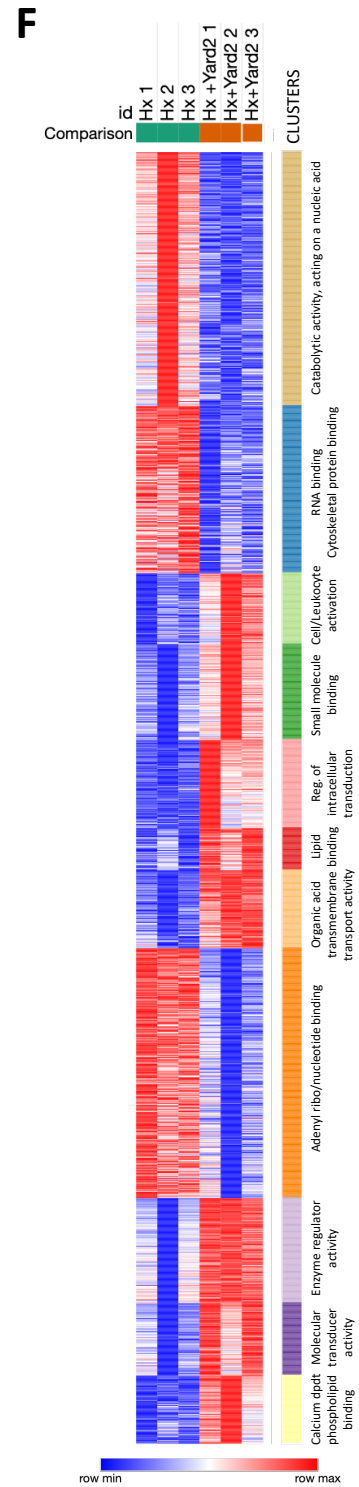

Suppl. FIGURE 3

Supplement: Supplementary file 3 — Supplementary Material 3. Figure 3. A and B, Heatmap (A) and the representative bar diagram (B) of some specific HIF-2 target genes in normoxia (Nx) compared to hypoxia (Hx) or hypoxia treated with Yard. 2. C, Heatmap of differentially expressed genes between normoxia (Nx) and hypoxia (Hx). The top molecular functions of the 11 clusters characterized are listed on the right. D, PCA plot of normoxia (Nx) and hypoxia (Hx) samples. Ellipses and shapes show clustering of the samples. E, Gene set enrichment map of RNA-Seq data comparing hypoxia (Hx) to normoxia (Nx) using “Cellular components”. F, Heatmap of differentially expressed genes between hypoxia (Hx) and hypoxia + Yard. 2 (Hx + Yard. 2). The top molecular functions of the 11 clusters characterized are listed on the right. [file 11658_2024_617_MOESM3_ESM.pdf]

A

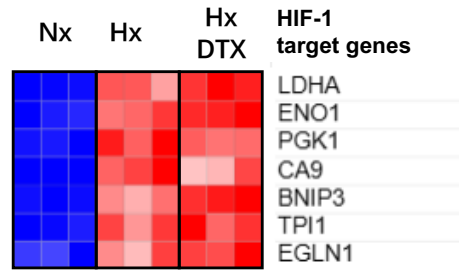

B

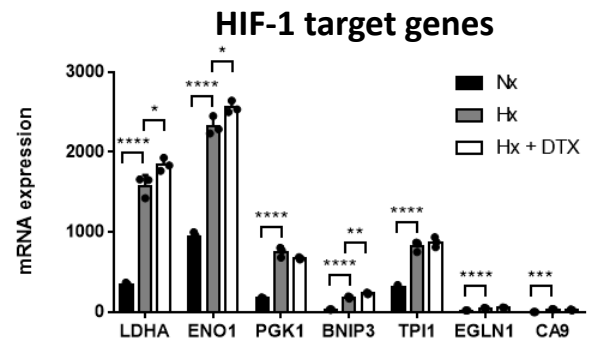

C

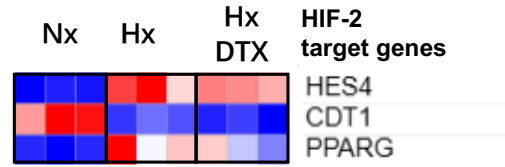

D

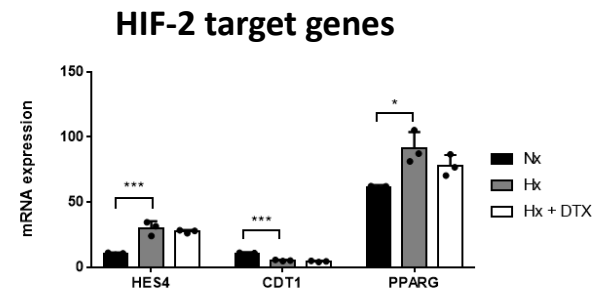

Suppl. FIGURE 4

Supplement: Supplementary file 4 — Supplementary Material 4. Figure 4. A and B, Heatmap (A) and the representative bar diagram (B) of some specific HIF-1 target genes in normoxia (Nx) compared to hypoxia (Hx) or hypoxia treated with Docetaxel (DTX). C and D, Heatmap (C) and the representative bar diagram (D) of some specific HIF-2 target genes in normoxia (Nx) compared to hypoxia (Hx) or hypoxia treated with Docetaxel (DTX). [file 11658_2024_617_MOESM4_ESM.pdf]
